# Development and validation of data quality rules in administrative health data using association rule mining

**DOI:** 10.1186/s12911-020-1089-0

**Published:** 2020-04-25

**Authors:** Mingkai Peng, Sangmin Lee, Adam G. D’Souza, Chelsea T. A. Doktorchik, Hude Quan

**Affiliations:** 1 Department of Community Health Sciences, University of Calgary, Calgary, Alberta Canada; 2 Analytics, Alberta Health Services, Calgary, Alberta Canada; 3 Centre for Health Informatics, University of Calgary, Calgary, Alberta Canada

**Keywords:** Association rule mining, Administrative data, ICD-10, Data quality assessment

## Abstract

**Background:**

Data quality assessment presents a challenge for research using coded administrative health data. The objective of this study is to develop and validate a set of coding association rules for coded diagnostic data.

**Methods:**

We used the Canadian re-abstracted hospital discharge abstract data coded in International Classification of Disease, 10th revision (ICD-10) codes. Association rule mining was conducted on the re-abstracted data in four age groups (0–4, 20–44, 45–64; ≥ 65) to extract ICD-10 coding association rules at the three-digit (category of diagnosis) and four-digit levels (category of diagnosis with etiology, anatomy, or severity). The rules were reviewed by a panel of 5 physicians and 2 classification specialists using a modified Delphi rating process. We proposed and defined the variance and bias to assess data quality using the rules.

**Results:**

After the rule mining process and the panel review, 388 rules at the three-digit level and 275 rules at the four-digit level were developed. Half of the rules were from the age group of ≥65. Rules captured meaningful age-specific clinical associations, with rules at the age group of ≥65 being more complex and comprehensive than other age groups. The variance and bias can identify rules with high bias and variance in Alberta data and provides directions for quality improvement.

**Conclusions:**

A set of ICD-10 data quality rules were developed and validated by a clinical and classification expert panel. The rules can be used as a tool to assess ICD-coded data, enabling the monitoring and comparison of data quality across institutions, provinces, and countries.

## Background

Administrative health data are generated at every encounter with the health care system, whether through a visit to a physician’s office or emergency department, or an admission to hospital [1, 2]. These data contain rich clinical and health service utilization information and have been widely used in epidemiological studies, disease surveillance, and health services research [3–5]. However, since the data are primarily collected for administrative or billing purposes, there are underlying concerns about whether they are suitable for other secondary purposes. For example, an international comparison of health system performance using a set of validated patient safety indicators on administrative health databases across 15 Organization for Economic Co-Operation and Development (OECD) countries showed inconsistent effect estimates due to issues of coding inconsistency and incompleteness across countries [6].

In administrative health data, patients’ specific conditions are often identified using a list of International Classification of Diseases (ICD) codes. The validity of ICD codes for identifying specific conditions depends on whether the condition contributes to health service use, and on where, when, and how data are collected [7, 8]. Therefore, data quality is an important issue for administrative health data and there is an urgent need for data quality assessment tools. Various data quality frameworks, such as the information quality framework from the Canadian Institute for Health Information (CIHI), have been developed to describe and assess data quality [9].

A practical definition for data quality is whether the data can be used for their intended purpose [10]. A dataset with high data quality should allow results observed in other studies to be reproduced. For example, Lewis et al. compared data quality between two electronic medical record (EMR) databases by evaluating whether the associations between diseases or between diseases and drugs can be reproduced as expected [11]. If the analysis results for the same study are consistent from two databases, the two databases are comparable in that respect. In drug safety surveillance, a set of drug-outcome pairs with negative (no effect) and positive (increased effect) associations was developed to check whether a dataset can reproduce drug-outcome associations as expected [12]. Similarly, data quality can be assessed by checking whether a set of expected associations can be observed in a dataset, and the degree of conformity to the expected associations reflects the data quality.

Our previous study proposed to use association rule mining to find coding associations in administrative health data and demonstrated that those association rules are useful for checking coding completeness and consistency [13]. The main objective of this study was to develop and validate a subset of clinically relevant coding association rules. In this study, we systematically applied the association rule mining method in Canadian re-abstracted inpatient data to develop a manageable set of coding association rules to be reviewed for coding and clinical validity through expert panel review. Our study does not aim to obtain a complete list of clinically valid association rules; rather, this work provides a data quality assessment tool for monitoring and comparing data quality of inpatient administrative healthcare databases using a particular set of clinically relevant association rules.

## Methods

### Data source

The Canadian hospital discharge abstract database (DAD) is a national database managed by CIHI, capturing administrative, clinical, and demographic information on discharges from acute care hospitals from all provinces and territories except Quebec [14]. The clinical information is coded using International Classification of Diseases, 10th revision, Canada (ICD-10-CA) codes, with up to 25 diagnosis codes per admission record. In Canada, ICD codes are assigned by health information management specialists (referred to as “coders”) through review of information documented by the physicians in patients’ health records following the coding standards developed by CIHI [15]. In this study, we used the CIHI re-abstracted DAD from the fiscal years of 2006–2011 and 2015 for rule development. The re-abstraction studies were part of CIHI’s data and information quality program to evaluate the quality of abstract coding. Re-abstraction of the DAD is a process involving recoding by external coding specialists of select charts that were previously coded by hospital coders [14]. Patient records for the re-abstraction study were selected based on a two-stage sampling process. First, acute care facilities with annual volume of > 1000 abstracts were sampled; then, patient records were sampled from the selected facilities. Re-abstracted data from CIHI can be deemed as the reference standard for coded data in Canada, as it is coded by highly trained coders. Coding reliability checks for external coders were conducted before the re-abstraction process.

### Data pre-processing

The ICD-10 classification system was developed by the World Health Organization (WHO) in 1992 and updated regularly thereafter [16]. Currently, more than 100 countries have adopted ICD-10 to report mortality and morbidity data. Some countries, including Canada, modify the codes to meet their specific needs by adding more specific codes. For example, ICD-10-CA adds 1 to 3 characters to the original ICD-10 codes to increase granularity in the specification of diseases. Similarly, the United States created ICD-10, clinical modification (CM) for billing purposes [15, 16]. ICD-10 codes have an alphanumeric format with a code size ranging from 3 to 7 characters depending on country. ICD-10 follows a hierarchical structure with the first three characters indicating category of diagnosis, the fourth digit added to indicate anatomic site, severity and other vital details, and the fifth to seventh digits as extensions added for different purposes by different countries. To ensure the generalizability of coding association rules, we mapped ICD-10-CA codes back to the original WHO version of ICD-10 codes.

There are 22 chapters in the ICD-10 codes. We only used the codes from chapter I to XVII in the rule mining process as codes from other chapters are related to symptoms and signs, causes of injury and poisoning, and health services uses.

### Rule mining process

For this study, we used an association rule mining (ARM) technique, a process of finding interesting associations or patterns hidden in a database, which we have described in previous work [13]. We used the Apriori algorithm, as implemented in the R package arules [17]. An association rule in the context of this paper is a relation of the form “X => Y”, where X and Y are sets of ICD-10 codes. We refer to X and Y as the left-hand side (LHS) and right-hand side (RHS) of the association rule respectively (they are also known in the literature as the antecedent and consequent of the rule respectively). When such a rule is discovered via a data mining procedure, it means that when a DAD record contains the codes in X, it is more likely to contain the codes in Y than would be expected by random chance. One metric that can be used for assessing the importance of an association rule is its support. The support count of a set of codes is the raw number of records containing all of the codes in the set, and the support is the proportion of records containing those codes. When evaluating an association rule X = > Y, one may be interested in the support of the LHS, i.e. P(X), the RHS, i.e. P(Y), or both jointly, i.e. P(X, Y). Another important metric for rule importance is its confidence, which is the probability that the RHS occurs given that the LHS occurs, i.e. P(Y|X). P(X,Y) is used as a measure of rule importance, support of LHS or P(X) quantifies the coverage of the rule, and confidence or P(Y|X) measures the reliability of the inference made by the rule.

The re-abstracted DAD was randomly split into training (70%) and validation (30%) data. Rule mining was conducted in four age categories (0 to 4, 20 to 44, 45 to 64, and ≥ 65), since patterns of disease conditions are age-dependent. No rules were identified for the age group 5–19 due to small sample size. When mining association rules, the results are affected by the choice of minimum thresholds for support count and confidence. If the thresholds are very high, only a small number of rules are expected, and it is unlikely that codes for rare conditions will be well represented. On the other hand, if the thresholds are too low, the number of rules generated will be too large for panel review to be feasible. To our knowledge, there are no applicable reference standards for these thresholds in the literature. Hence, after an iterative process of experimenting with thresholds to balance these competing concerns, we set a confidence threshold of ≥0.05. Although we wanted the rules to have a minimum support count of 20, there was no need to explicitly set a threshold for the support, as the confidence of a rule P(Y|X) must always mathematically be higher than its support P(X,Y), and because the number of DAD records was greater than 400 for each age group considered, this implies that the support count of any rule satisfying the confidence threshold would necessarily be higher than 20. Two sets of rules, one each at the three-digit and four-digit levels of ICD codes, were developed.

Two rules are considered nested if they have the same RHS, and the LHS of one rule is a subset of the LHS of the other. For example, the two rules X = > Y and {X, Z} = > Y are nested. If the difference in confidence between two nested rules is less than 0.1, we only kept the simpler rule.

The chi-squared test or Fisher’s exact test were applied to the rules to assess the statistical association of the left and right hand side of the rules. Rules were deemed non-significant and excluded if the false-discovery-rate-adjusted *P* value was < 0.05 [18].

The rules were then applied to the validation data and the confidence of each rule on the validation data was calculated. Rules were excluded if the difference in rule confidence between the training and validation data was larger than 5%. Rules reflecting dagger-asterisk coding systems to indicate both the etiology (dagger codes) and manifestations (asterisk codes) of a disease were also excluded [15].

### Rule review by an expert panel consisting of physicians and coding specialists

We used a modified Delphi panel process to review the association rules identified in step 1. Panellists were asked to rate the appropriateness of rules from the clinical perspective and the extent to which it is potentially suitable as a data quality rule [19]. A meaningful clinical association might reflect that one condition is a direct cause for another, or one condition is a risk factor for another condition; or one condition co-occurs with another condition because of similar risk factors.

**Fig. 1:**
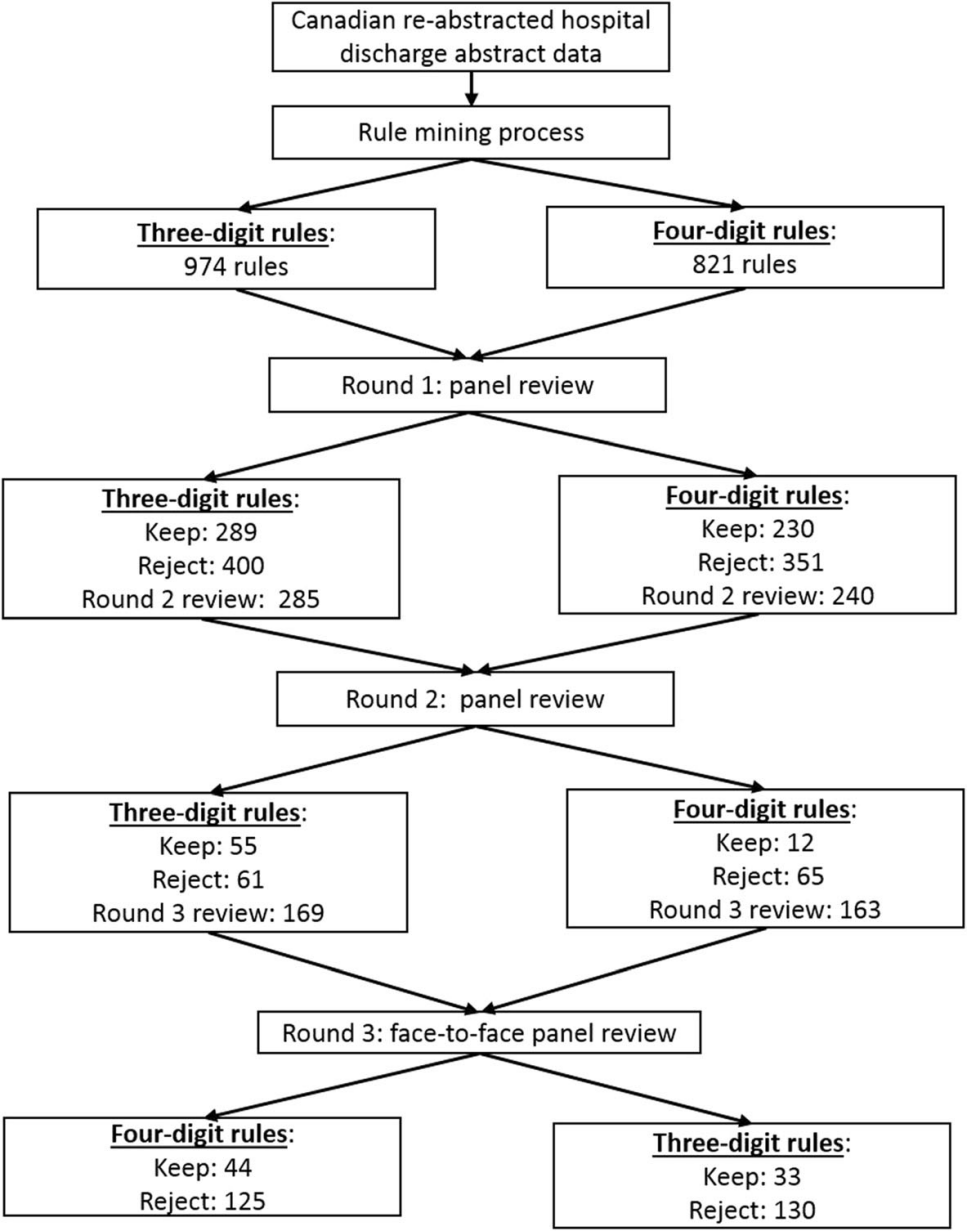
Diagram for rule development process

Five physicians from the clinical areas of paediatrics, internal medicine, geriatrics, endocrinology, and family medicine, and two coding specialists from CIHI were invited to review the rules. We used a three-step rating process with two rounds of remote review conducted independently to identify the association rules that were disagreed upon (Fig. 1). This was followed by a two-day face-to-face panel review to discuss reasoning for disagreement and come to a consensus decision for association rules to include. We adapted the RAND appropriateness method, which involved rating clinical scenarios on a nine-point scale, to determine the clinical and coding appropriateness of our association rules [19]. To ensure consistency in the panellist answers in reviewing the association rules, we pilot-tested the review on the first five association rules and discussed any confusions or misunderstandings of the process.

Specifically, we asked participating panellists the following question: “Please rate the appropriateness of this candidate rule from the clinical perspective (whether the rule captures a meaningful or expected clinical association), and thus the extent to which it would be appropriate as a data quality rule (DQR)”. Their response options included: 1, 2, 3 = not supported by clinical evidence and thus not appropriate as a DQR; 4, 5, 6 - uncertain regarding its clinical association and thus uncertain of merit as a DQR; 7, 8, 9: supported by clinical evidence and thus appropriate as a DQR. We also provided an option for “Unknown”, if a panelist believed that they did not have enough evidence to provide the answer.

Drawing upon RAND definitions of agreement of appropriateness ratings, we considered there to be panel agreement when 4 or all of the physicians’ ratings fell within the same 3-point zone of appropriateness (i.e. 1–3, 4–6, or 7–9) and no coders raised any potential coding issues. Rules without such agreement after two rounds of reviews were discussed at the face-to-face meeting and re-rated after discussion, whereas those with agreement prior to the meeting were not discussed unless a panellist expressed desire to discuss a particular diagnosis.

### Assessing the data quality of hospital discharge data from Alberta, Canada

Rules were applied to DAD data from Alberta acute care hospitals with annual volume of over 1000 records between April 1, 2013 and March 31, 2014. Rule confidence and support were calculated at the hospital levels. Hospitals were divided into comparable groups based on hospital types and volumes: teaching hospitals, large community hospitals, etc. Bias and variance were defined and calculated to assess hospital data quality. High quality data should have low bias (close to the golden or reference standard) and low variance (consistent across institutions). We used the confidence from the CIHI re-abstracted data as the reference standard for bias calculation. 95% confidence intervals for rule confidence at the hospital level were calculated using the Wilson method and range of confidence intervals was calculated as the difference between lower and upper bounds [20]. Bias for each rule was defined as the sum of (1 minus range) × absolute confidence difference between Alberta hospitals and CIHI data. To calculate the variance, we first calculated the 95% confidence intervals of pairwise differences between hospitals within the same group using the studentized range distribution with a score statistic and range of confidence intervals is calculated [21]. Variance was calculated as the sum of (2 minus range) × absolute confidence difference between hospitals for each rule. The rules were ranked based on bias and variance respectively for different hospital groups. The top 5 rules with highest bias or variance for teaching hospitals is presented here.

## Results

Association rule mining was applied to the 30,628 re-abstracted records with a median of three (interquartile range (IQR): two to four) ICD codes. After removing the nested and non-significant rules, there were 974 rules at the three-digit level and 821 rules at the four-digit level for panel review. After the panel review, there were 388 rules at the three-digit level and 275 rules at the four-digit level (see Table 1). The plurality of the rules was for the age group ≥65 years (46.4% of the three-digit rules and 51.3% of the four-digit rules). Three-digit rules included 82 unique codes on the right-hand side and 107 on the left-hand side, while four-digit rules included 93 unique codes on the right-hand side and 84 on the left-hand side. Overall confidence and support for the three-digit rules were slightly higher than for the four-digit rules (see further details in Table 1).

**Table 1:** Distribution of rules after the panel review

	Three-digit rules	Four-digit rules
N	388	275
Age group		
0 to 4	52 (13.4%)	43 (15.6%)
20 to 44	82 (21.1%)	49 (17.8%)
45 to 64	74 (19.1%)	42 (15.3%)
≥65	180 (46.4%)	141 (52.3%)
Number of unique ICD codes		
Right hand side of rules	82	93
Left hand side of rules	107	84
Both sides of rules	114	106
Confidence of rules		
0.05 to 0.2	131 (33.8%)	146 (53.1%)
0.2 to 0.5	158 (40.7%)	89 (32.4%)
≥0.5	99 (25.5%)	40 (14.5%)
Support of rules		
0.2 to 1%	205 (52.8%)	165 (60.0%)
1 to 3%	136 (35.1%)	69 (25.1%)
≥3%	47 (12.1%)	41 (14.9%)

**Fig. 2:**
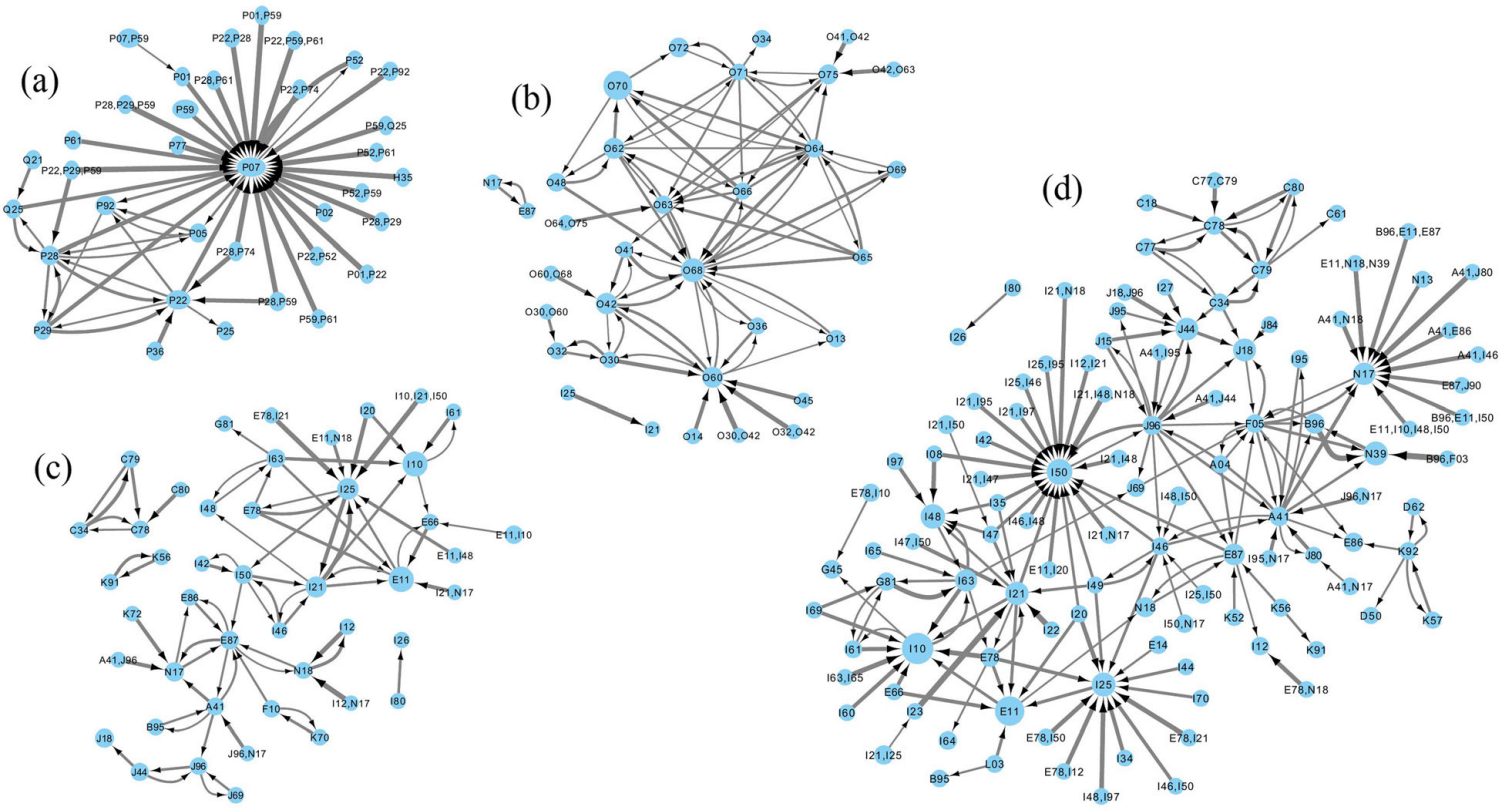
Three-digits rules at the four age groups: **a**) 0–4; **b**) 20–44; **c**) 45–64; **d**) ≥ 65

**Fig. 3:**
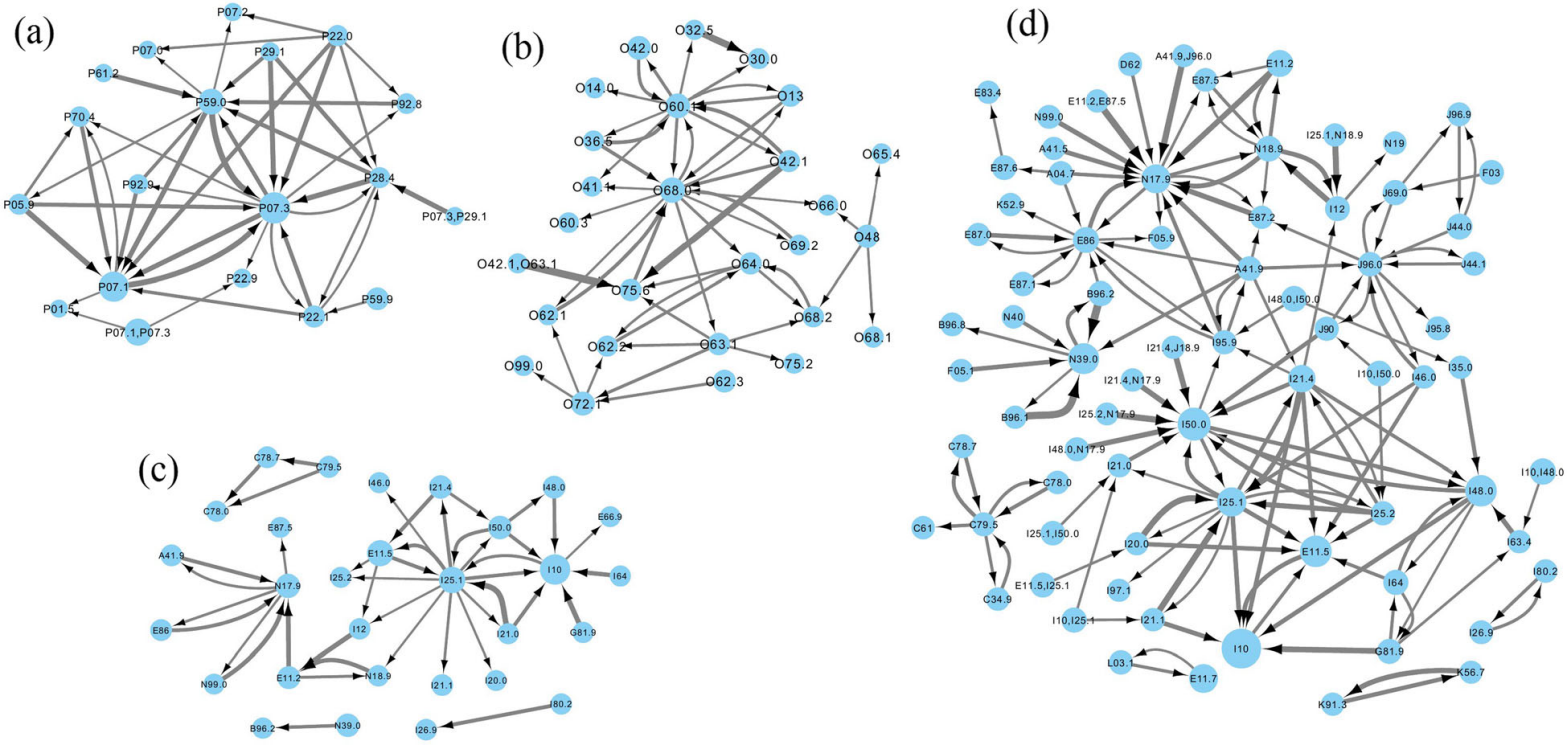
Four-digits rules at the four age groups: **a**) 0–4; **b**) 20–44; **c**) 45–64; **d**) ≥ 65

Rules captured relevant clinical associations at their corresponding age groups. For the age group 0 to 4 (Fig. 2 (a) and Fig. 3 (a)), all the codes in the four-digit rules were from the chapter for conditions originating in the perinatal period. The codes that appeared in the most rules (highest degree centrality) were P07.1/P07.3: disorders related to short gestation and low birth weight, P28.4: apnoea of newborn/prematurity and P59.0: neonatal jaundice associated with preterm delivery. The three-digit rules also identified a few associations with codes from other chapters, such as H35 (retinal disorders) and Q21/Q25 (related to the circulatory system).

For the age group 20 to 44 (Fig. 2 (b) and Fig. 3 (b)), all the codes in the four-digit rules were from the chapter about pregnancy, childbirth, and puerperium. The most commonly used codes were related to complications of labour and delivery. Those included O60.1: preterm spontaneous labour with preterm delivery, O68.0: labour and delivery complicated by fetal heart rate anomaly, etc. Three-digit rules included codes from ischaemic heart disease (I21: acute myocardial infarction and I25: chronic ischaemic heart disease), E87: disorders of fluid, electrolyte and acid-base balance, and N17: acute renal failure.

For the age group 45 to 64 (Fig. 2 (c) and Fig. 3 (c)), the majority of the codes used in the rules were from chapters about diseases of the circulatory system, endocrine/nutritional/metabolic diseases, and diseases of the genitourinary system. Rules in the age group ≥65 (Fig. 2 (d) and Fig. 3 (d)) included codes from all other chapters except for the two chapters whose codes appeared in the rules for age groups 0 to 4 and 20 to 44. Rules for the age group ≥65 included codes from more different chapters than the other age groups, and displayed a more complex association structure. This was likely due to high prevalence of conditions and complexity of patients’ conditions.

**Fig. 4:**
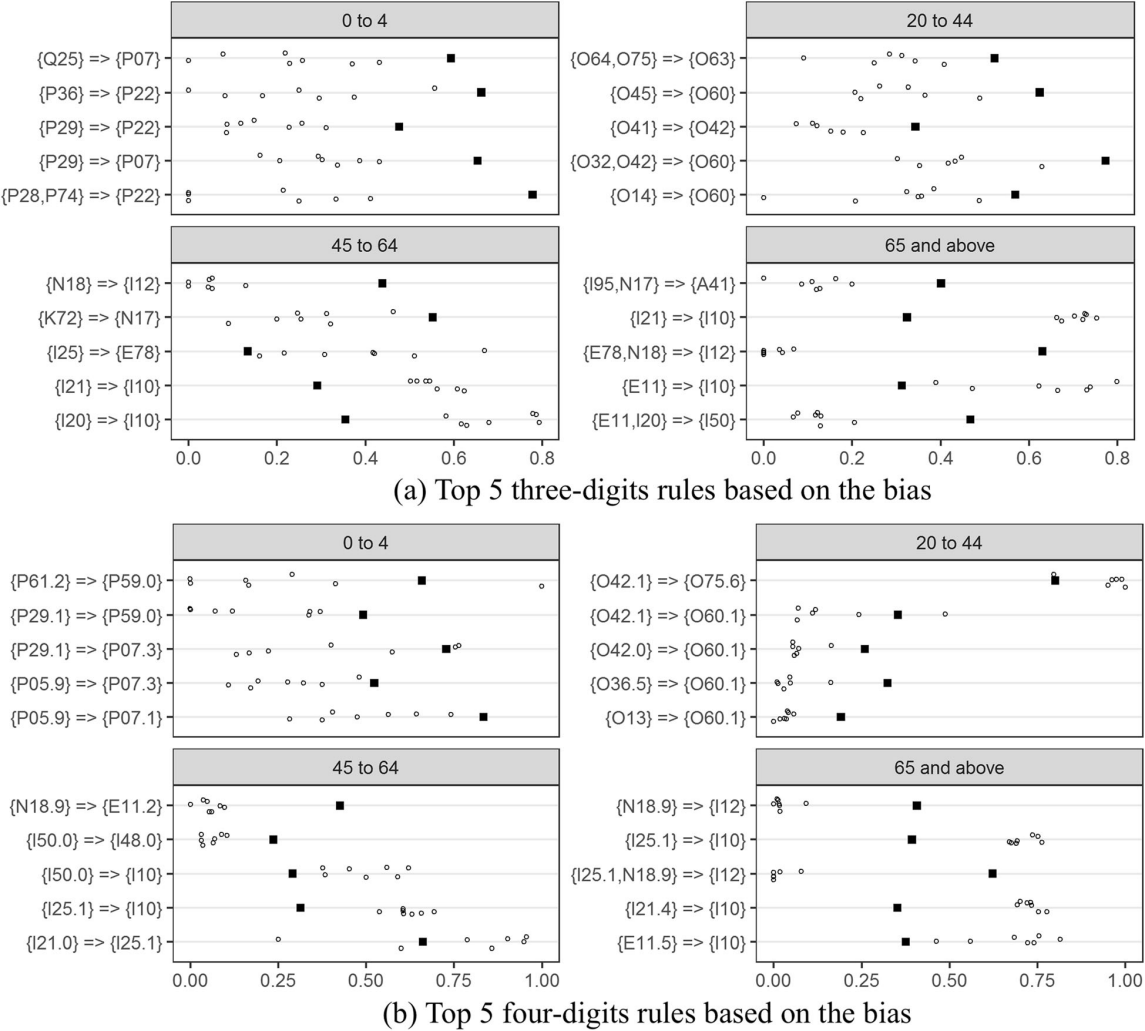
Top 5 rules based on bias: square for CIHI and circles for Alberta teaching hospitals

**Fig. 5:**
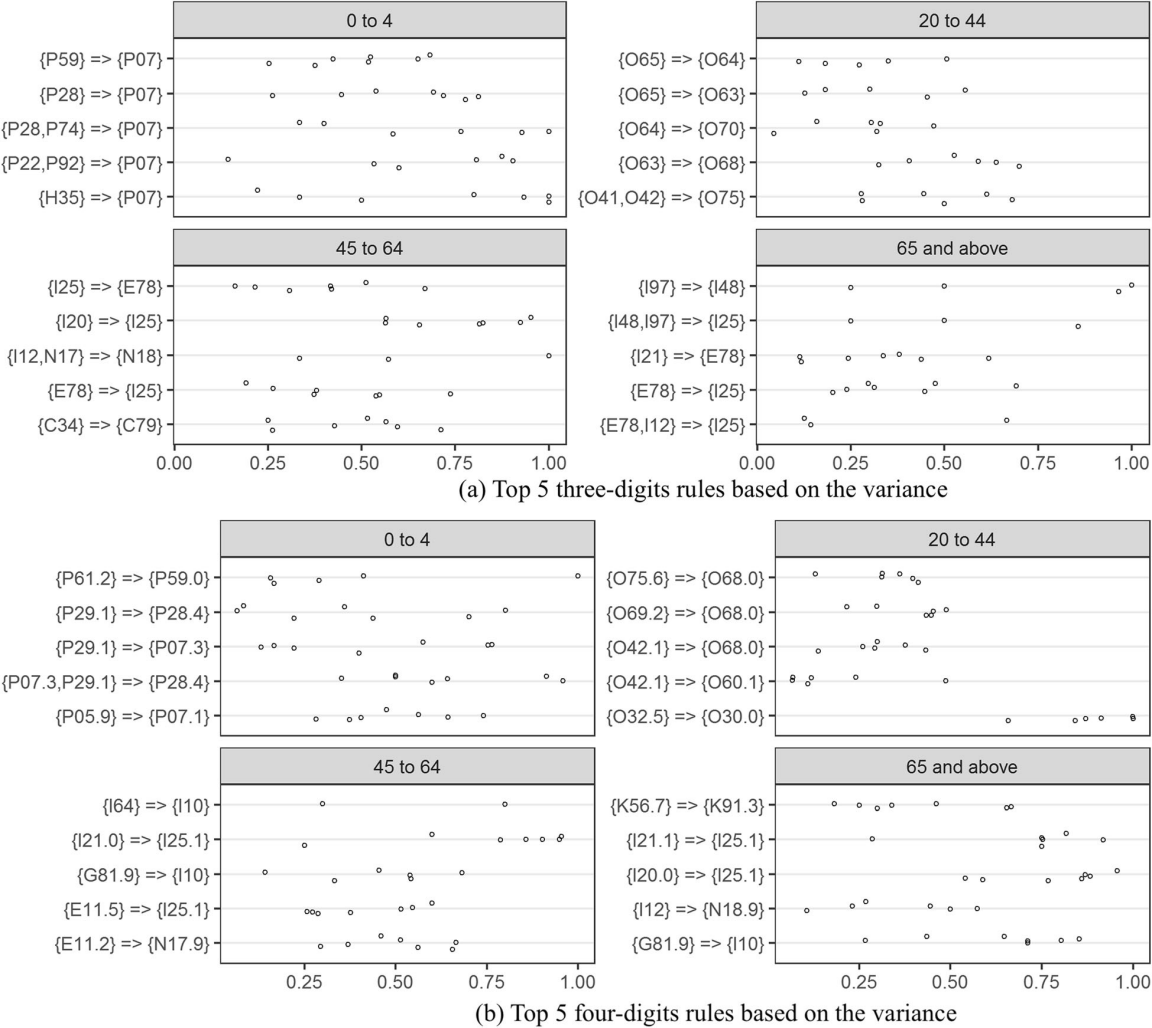
Top 5 rules based on variance: circles for Alberta teaching hospitals

Rules were then applied to assess the quality of 2013 Alberta DAD data. For the top 5 rules with high bias at the three and four digits (Fig. 4), Alberta data generally had lower rule confidences than CIHI re-abstracted data except for three-digit rules involving the codes I10: essential hypertension or E78: disorders of lipoprotein metabolism and other lipidaemias on the right hand side, and four-digits rules with codes O75.6: delayed delivery after spontaneous or unspecified rupture of membranes, I25.1: atherosclerotic heart disease, and I10. Alberta mandatorily codes I10 for surveillance purpose, which leads to more complete capture of hypertension and high confidence for the I10 related rules than other provinces in Canada. Some codes, such as I12: hypertensive renal disease for the age groups 45 to 64 and ≥ 65, and E11.2: type 2 diabetes with renal complications, were systematically under-coded in Alberta as indicated by very low confidence across all hospitals. A variance plot (Fig. 5) shows the differences of rules confidences between Alberta hospitals. The bias and variance measures identified different sets of deviations from expected associations that could potentially indicate data quality issues.

## Discussion

Through the analysis of the nationwide re-abstracted hospital discharge data and an extensive modified Delphi process, this study has developed a comprehensive set of ICD-10 diagnosis code rules that can be used for data quality assessment at the three-digit and four-digit levels for four different age groups. Three-digit rules covered more codes and displayed more complex association networks compared to four-digit rules. Rules for age groups 0 to 4 and 20 to 44 captured conditions concerning the perinatal period and conditions occurring during pregnancy, childbirth, and the postpartum period, respectively. Rules for age groups 45 to 64 and ≥ 65 captured a wider range of conditions. Finally, the application of the association rules was demonstrated by assessing data quality across 27 Alberta hospitals. These rules can be used in other contexts for comparisons between hospitals, provinces and countries.

Hospital administrative health data captures rich information on each hospitalization using standardized terminologies. However, the quality of coded hospital administrative data for research and reporting purposes has been widely debated since it is primarily collected for administrative/billing purposes and aims to capture clinically significant diagnoses that require treatment or the use of clinical resources [15]. Validation studies have shown substantial variations in validities of ICD codes. Among the 32 conditions included in the Charlson and Elixhauser comorbidities, sensitivity ranged from 0.127 for weight loss to 0.808 for metastatic cancer and positive predictive value ranged from 0.32 for blood loss anemia to 1 for AIDS/HIV [8]. It should be emphasized that the specificities of ICD codes were generally very good, with most conditions having specificity close to 0.99.

The coding process is very complex, with many possible opportunities for inconsistency and incompleteness [22]. Current coding standards only stipulate mandatory coding of clinically significant conditions [15]. Other barriers to high quality of coding include clinical documentation, variability in interpretation, and high quota expectations [23, 24]. Differing documentation practices between physicians and between hospitals result in varying levels of details in patient charts and variability in the documents available for data abstraction, which might cause issues of incompleteness [25]. Variability in chart interpretation and coding knowledge between coders can also result in coding inconsistency [23]. Pressure arising from high coding quotas can also lead to focus on productivity over quality, resulting in variations in coding completeness. Therefore, deciding how to assess coding consistency and completeness is a high priority for quality assessment of coded administrative health data.

Several conceptual data quality frameworks have been developed in Canada and other countries [26, 27]. For example, CIHI created an information quality framework and used five dimensions (relevance, accuracy and reliability, comparability and coherence, timeliness and punctuality, accessibility and clarity) to describe and assess quality relative to users’ needs [9]. Other researchers have harmonized data quality assessment terminologies and frameworks to enable data owners/users, patients, and policy makers to evaluate and communicate data quality findings in a well-defined manner with a shared vocabulary [28]. However, there is always a gap that remains between data quality terminology/frameworks and actionable tools for data quality assessment and reporting. Our study provides comprehensive lists of actionable data quality rules specifically designed for ICD coded data. Application of the rules can be viewed as a “slice and dice” process of breaking data into blocks defined by age groups, and examining the information from different clinical scenarios. Each rule examines a particular clinical scenario within a specific age group.

This tool has several advantages. First, rules were developed from a data-driven perspective and reflect the real practice of coding. As association rule mining may identify spurious rules resulting from randomly occurring correlations between codes, another strength of our study was that the clinical validity of the rules was guaranteed through the expert panel review process. The rules included over 100 different ICD codes at the three- and four-digit levels respectively, and covered over one third of diagnosis codes being used in practice. Furthermore, the rules provide a cost-effective means for conducting data quality assessments. Re-abstraction studies involve the recruitment of trained coding specialists to recapture the data following the same coding standard. The high cost restricts the number of records being re-abstracted and limits its scale of application. Detailed discussion about how to potentially apply rules for data quality assessment can be found in our previous publication [13].

We proposed and defined measures of bias and variance to identify potential data quality issues. Bias reflects the degree of difference of a data source compared to a reference standard. High bias might indicate high levels of under-coding or over-coding for one data source as compared to the reference standard. Variance reflects the degree of variability between different data sources. High variance indicates high levels of coding inconsistency across data sources. Bias and variance are weighted sums of absolute confidence difference. They assign high weights if the confidence interval is narrow. Bias and variance plots could be an intuitive measure to assess coding incompleteness and inconsistency and a potential measure indicator for data quality. Use of bias and variance measures could provide directions for data quality improvement.

Our study also has certain limitations. First, association rule mining is a type of unsupervised learning with several tuning parameters (e.g. threshold of support and confidence) for rule development. Although we have used the Apriori algorithm for our association rules implemented in implemented in arules package, other researchers with large datasets may consider using other, potentially more efficient association rule mining algorithms for rule development [29, 30]. We have implemented several methods to filter rules to ensure the uniqueness and robustness of rules to minimize the number of rules for panel review based on domain knowledge and statistical assessment, and may therefore have excluded useful rules during the rule cleaning process. Second, there is a need for further validation of the association rules against chart review or prospective case reviews. A more complete version of coded data is needed to establish the reference standard of rule confidences. We expect the rule confidences would generally increase due to common issues of under-coding in coded data. Third, rules were developed using a data-driven approach, and their completeness depends on the quality of re-abstracted data. Rules involving certain conditions might be missed due to errors in the re-abstracted data, as well as because the data reflects Canadian coding practices that aim to balance completeness with efficiency, rather than focusing solely on comprehensiveness. Fourth, rules only assess data quality at the aggregate level. Deviation from a rule by a single institution does not necessarily indicate an error. We did not provide a specific acceptability threshold for monitoring the quality of data for use because we have only selected a subset of acute care facilities in Alberta with certain characteristics. The association rules are soft, probabilistic rules that may have different appropriate thresholds for different sets of health care system characteristics. The variances between the re-abstracted data of the DAD from the original data from each of the institutions in Alberta, may be due to differences in specific coding standards and requirements of that institution. Thus, further investigation of rule confidence discrepancies and external validation are still required to gain a better understanding on how to set appropriate thresholds for data quality association rules.

## Conclusion

In conclusion, we have developed a set of data quality rules through the association rule mining. The clinical and coding validity of rules were guaranteed through the panel review process. Rules covered interesting clinical scenarios at the different age groups. Bias and variance can be used to assess coding incompleteness and inconsistency between hospitals. The resulting work has great potential to monitor and compare ICD coded data across institutions, provinces and countries.

## Data Availability

The data are available from Alberta Health Services and the Canadian Institute of Health Information, subject to their approval.

## Abbreviations

ARM: Association rule mining
ICD-10: International Classification of Diseases, 10th revision
CIHI: Canadian Institute of Health Information
CM: Clinical Modification
DAD: Discharge Abstract Database
DQR: Data quality rule
IQR: Interquartile range
LHS: Left-hand side
OECD: Organization for Economic Co-operation and Development
RHS: Right-hand side
WHO: World Health Organization
